# Application of machine learning models based on decision trees in classifying the factors affecting mortality of COVID-19 patients in Hamadan, Iran

**DOI:** 10.1186/s12911-022-01939-x

**Published:** 2022-07-24

**Authors:** Samad Moslehi, Niloofar Rabiei, Ali Reza Soltanian, Mojgan Mamani

**Affiliations:** 1 Department of Biostatistics, School of Public Health, Hamadan University of Medical Sciences, Hamadan, Iran; 2 Modeling of Noncommunicable Diseases Research Center, School of Public Health, Hamadan University of Medical Sciences, Street of Shahid Fahmideh, P.O. BOX: 6517838736, Hamadan, Iran; 3 Brucellosis Research Center, Hamadan University of Medical Sciences, Hamadan, Iran

**Keywords:** Machine learning, CART, C4.5, C5.0, Logistics model tree, COVID-19, Classification

## Abstract

**Background:**

Due to the high mortality of COVID-19 patients, the use of a high-precision classification model of patient’s mortality that is also interpretable, could help reduce mortality and take appropriate action urgently. In this study, the random forest method was used to select the effective features in COVID-19 mortality and the classification was performed using logistic model tree (LMT), classification and regression tree (CART), C4.5, and C5.0 tree based on important features.

**Methods:**

In this retrospective study, the data of 2470 COVID-19 patients admitted to hospitals in Hamadan, west Iran, were used, of which 75.02% recovered and 24.98% died. To classify, at first among the 25 demographic, clinical, and laboratory findings, features with a relative importance more than 6% were selected by random forest. Then LMT, C4.5, C5.0, and CART trees were developed and the accuracy of classification performance was evaluated with recall, accuracy, and F1-score criteria for training, test, and total datasets. At last, the best tree was developed and the receiver operating characteristic curve and area under the curve (AUC) value were reported.

**Results:**

The results of this study showed that among demographic and clinical features gender and age, and among laboratory findings blood urea nitrogen, partial thromboplastin time, serum glutamic-oxaloacetic transaminase, and erythrocyte sedimentation rate had more than 6% relative importance. Developing the trees using the above features revealed that the CART with the values of F1-score, Accuracy, and Recall, 0.8681, 0.7824, and 0.955, respectively, for the test dataset and 0.8667, 0.7834, and 0.9385, respectively, for the total dataset had the best performance. The AUC value obtained for the CART was 79.5%.

**Conclusions:**

Finding a highly accurate and qualified model for interpreting the classification of a response that is considered clinically consequential is critical at all stages, including treatment and immediate decision making. In this study, the CART with its high accuracy for diagnosing and classifying mortality of COVID-19 patients as well as prioritizing important demographic, clinical, and laboratory findings in an interpretable format, risk factors for prognosis of COVID-19 patients mortality identify and enable immediate and appropriate decisions for health professionals and physicians.

## Background

With the onset of the COVID-19 pandemic, disruptions and adverse factors in the livelihoods of people have occurred that over 576 million people in the world have lost their lives due to this disease and its mortality rate is still high in some countries [1]. Many studies have been done since the beginning of the disease, and each has used different methods to investigate the mortality rate and its causes. In the research conducted in Babol, Northern Iran, from February to April 2020, out of 557 patients admitted, 121 died [2]. In another study conducted in Tehran, Iran, from March 2020 up to December 2020, out of 205,654 patients admitted, 20,472 died [3]. In East Azerbaijan Province, Iran, from the outbreak up to May 2021 (before vaccination), out of 18,079 confirmed cases, 4390 died [4]. Based on the reported results of WHO, from February 2020 to, July 2021, out of 3,871,008 confirmed cases, 90,630 died in Iran [1].

COVID-19 infection is considered to be the most serious infection in the world with the most common symptoms being fever, fatigue, and cough. The severity of the disease varies from person to person, such as shortness of breath and dysfunction of internal organs [5]. The diagnosis of COVID-19 is not based solely on the diagnosis of clinical symptoms such as fever or cough, and various clinical and laboratory biomarkers related to viral infection can be helpful in the treatment of this disease for physicians and clinicians [6]. Identification of clinical biomarkers that are effective in the progression of the disease to severe complications and even death of patients is significant in the treatment of this disease. Some biomarkers may be involved in the mechanisms of viral infection and cell and organ damage [7]. Influence of some demographic characteristics such as age, sex, history of diabetes and blood pressure, and some routine blood and biochemical tests, including white blood cells, lymphopenia, C-reactive protein, lactate dehydrogenase (LDH), creatine kinase (CPK), hemoglobin, Hematocrit, lymphocyte and neutrophil count, platelet count, prothrombin time and D-dimer were reported to be significant in association with COVID-19 disease severity [7–9].

Various machine learning methods have been utilized to classify and predict deaths due to COVID-19 disease, including random forest and decision trees [10–13], however, in these studies, no interpretation of the features and the relationship between changes in these features in patients' mortality has been examined and only classification indices are provided [14–17]. Since in addition to identifying the most important features and finding an accurate model in classifying patient mortality, it is also important to find the relationship between features and patient mortality.

One of the methods of machine learning is interpretable decision trees, which are considered classification techniques in data mining [11]. One of the most popular usages of decision trees is to display the results as a simple decision tree algorithm that is easy to interpret for most researchers. Because these trees can show the structure of decisions in the classification process, which are known as white-box models [18]. The Decision trees have capabilities such as non-parametric adjustment and control of heterogeneous data and can classify consecutive data in the best way, and if features are not normalized and scaled, they are also capable. Also, the structure of decision trees requires less execution time in data classification compared to other machine learning classification techniques [19]. There are several different approaches to decision trees, including the LMT, C4.5, C5.0, and CART trees, in a variety of research areas such as basic science studies [20], medicine [21], and classification images [22] have been utilized. The random forest is a conventional machine learning algorithm for solving complex problems which is one of the supervised learning methods and its structural model is based on the tree and is used in issues such as classification and regression. The random forest consists of several trees with different patterns from a series of training datasets, and the accuracy of predicting the datasets is calculated from the average of the trees. Often, this algorithm is used to find the importance of influential features in the response [23].

The Iterative Dichotomiser 3 (ID3) tree algorithm is developed using the information gain split criterion proposed by Quinlan in 1986. Based on the initial ID3 tree structure, improved algorithms from this tree were also proposed. Meanwhile, the C4.5 tree algorithm was introduced by Quinlan in 1993. In C4.5 tree, an expanded information gain criterion called information gain ratio (IGR) is used, which is suitable for using features with a large number of samples, to reduce defects in the ID3 tree. Pruning a tree is one of the benefits of decision trees that C4.5 has [24]. For large-scale datasets, new decision tree algorithms were introduced for classification and prediction, such as C5.0 and CART, which the C5.0 algorithm is very similar to the C4.5 tree. This algorithm has advantages and attributes such as missing data management and pruning method. Hence, the main application of decision trees is to create a training flowchart that can be used to classify or identify a class or value of a target variable based on decision rules learned from previous data (training data) [19]. Other available trees that can classify responses is LMT. This tree is obtained by combining two methods of logistic regression and decision tree algorithm which is used in binary response classification [25].

This cohort study aimed to identify the important features and find their relationship with patient mortality and provide a model with appropriate accuracy in classifying patient mortality. To achieve this, in the first step, important features were identified by the random forest method. In the second step, the LMT, C4.5, C5.0, and CART decision tree models were developed for classifying the COVID-19 mortality based on demographic characteristics, clinical, and laboratory findings of individuals selected in the first step. In the third step, accuracy, recall, and F1-score criteria were employed to evaluate the performance of these models and select the best. At last, the best decision tree employed and its interpretability flowchart are drawn, and the ROC curve and the AUC values are reported.

## Methods

### Study design and dataset

In this retrospective study, information of demographic characteristics, clinical and laboratory findings of 2470 patients with COVID-19 admitted to Sina (Corona treatment center) and Besat hospitals in Hamadan, west of Iran from February 2020, to July 2021, were collected from patients’ medical records. In this study, patients with positive real time reverse transcriptase polymerase chain reaction (RT-PCR) on samples from upper respiratory nasopharyngeal swabs were enrolled to the study.

Demographic characteristics i.e. age, sex, marital status, location, smoking, compromised immune system (C.I.S), renal insufficiency, diabetes, hypertension, cancer, Hematologic disorders, Cardivascular disease, lung diseases, Hepatic failure, and neurological diseases as well as laboratory biomarkers i.e. erythrocyte sedimentation rate (ESR), blood urea nitrogen (BUN), blood sugar (BS), CPK, Serum glutamic-oxaloacetic transaminase (SGOT), thromboplastin or partial thromboplastin time (PTT), platelets (Plat), sodium (NA), LDH, and: polymorphonuclear (PMN) were collected from the time of patients’ discharge. The outcome variable in this study was considered a binary status, dead = ‘1’ or recovered = ‘0’, where 1853 and 617 patients were recovered and dead, respectively.

Before performing any analysis, the data were preprocessed. An individual is removed from the dataset if there is at least one missing record in his/her features. It is important to note that the mechanism of missingness was examined before removing the missing data, and since the mechanism of missingness was completely at random, their removal would not lead to a bias in the results. If the individual features had outlier and illogical values, the individual information was removed from the study. Finally, after the two steps mentioned, the final dataset without any missing or outlier values was prepared for use in the next steps.

**Fig. 1:**
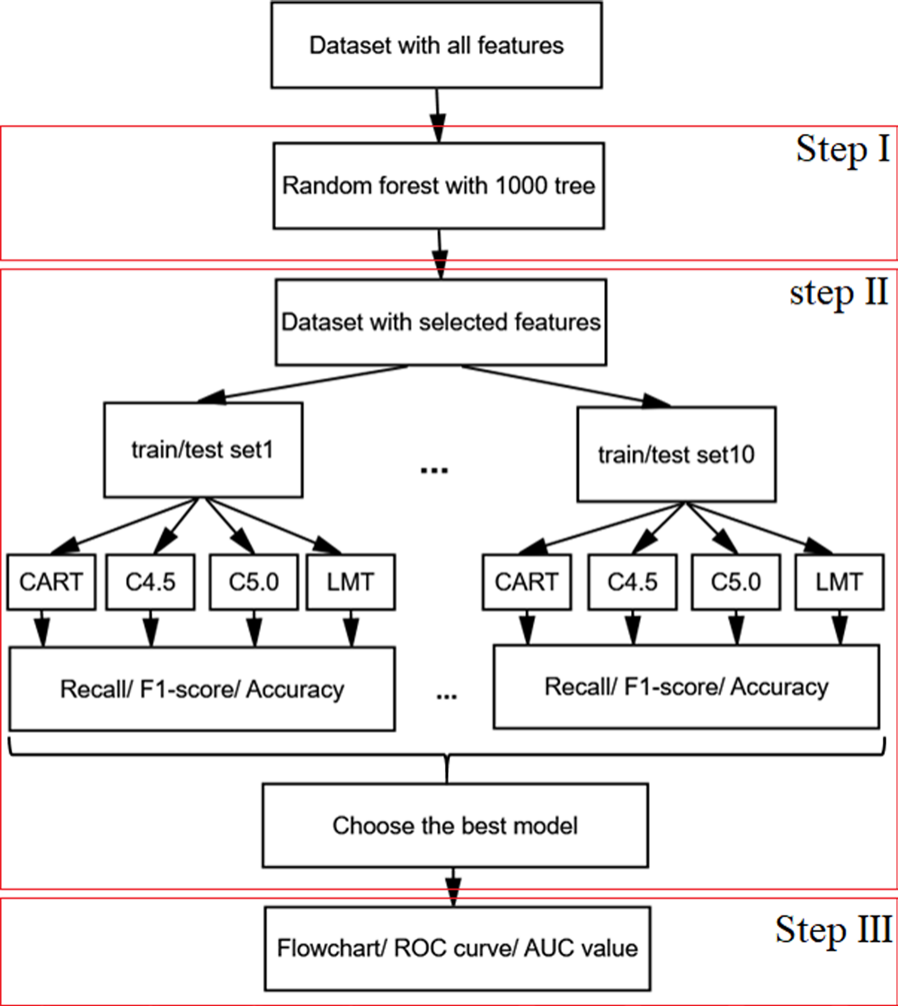
The process of COVID-19 classification mortality

The method of this study is divided into three steps consisting of feature selection, model comparison and selection of the best model, and interpretation. The steps are as follows and the Fig. 1 illustrates the process:

(I) Due to a large number of features studied and also the identification of important features for interpretable flowcharts, the random forest algorithm, which is one of the most common approaches to identify important features, was used. In this study, the random forest algorithm was used to generate 1000 tree and select the Gini criterion to calculate the importance of each feature. Based on the values obtained from the relative importance of the features, the third quartile was considered as a cut-point for selecting features, which was 6. Features with relative importance greater than 6% were selected for subsequent analysis. (Feature selection)
(II) To assess the performance of classification trees (LMT, C4.5, C5.0, and CART trees), first, the dataset is randomly divided into two subsets of training and test data. To achieve better results and reduce the randomization effect of the splitting test/train dataset, this procedure done 10 times. In fact, 10 separate datasets (include test and train data) are used separately for training the classification trees. After the training process, the performance of the model is evaluate based on the test dataset. Then, the evaluation indices (recall, accuracy, and F1-score) for each tree were calculated and the final result was reported by calculating the mean of these indices. The best tree is the one with maximum value index. (Model comparison and selection of the best model)
(III) The flowchart and ROC curve of the tree that was selected in step II will be drawn and interpreted. The AUC value also reported. (Interpretation)

All mathematical theories of the implementation steps, including the trees used, the classification process, and the evaluation indices are described in detail below.

### Decision tree

The decision tree is a directional tree that consists of several nodes. The first node of a tree is called the root node, which has no input. Other nodes have an input and an output, which is known as an internal node. Also other nodes can only have one input that will call as a leaf node or decision node. In the decision tree, the number of samples is divided into two or more sub-samples by internal nodes using a specific probability index such as entropy and Gini. Finally, each end node or leaf is assigned to the class that has the most ideal response values. There are several decision tree algorithms such as C4.5, C5.0, CART and logistics tree for discrete and continuous features [26].

### Logistic model tree (LMT)

In the logistics model, the information gain criterion is used to divide the samples into two subsets. However, in the LMT, a logistic regression model is fitted for each tree node, which is done using the LogitBoost algorithm. In other words, it models the posterior class probabilities for several classes, ${\text{G}}_{{\text{j}}} ;\;\;j = 1, \ldots ,J$, and estimates the maximum value of this probability. For an arbitrary feature *X* of class *J*, the LMT in terms of class $J - 1$, the logarithm-odds is as follows:

$$\log \left( {\frac{{P\left( {G = j|X = x} \right)}}{{P\left( {G = J|X = x} \right)}}} \right) = \beta_{j}^{T} x ,\quad j = 1, \ldots ,J - 1$$

where parameter $\beta_{j}$ is the effect of feature *X* in class *j*. The LMT is pruned using the CART algorithm and utilizes cross-validation to find the number of LogitBoost iterations to prevent the tree from over-fitting. The LogitBoost algorithm uses logistic regression of fitted least squares for each class [25].

### C4.5 and C5.0 decision tree

In C4.5 tree, splitting stops when the number of samples is less than a specified threshold value. Tree pruning is done according to the error criterion after the growth stage. The branches that are the least precise in dividing the tree are dropped and the leaf nodes are replaced. In these trees, the outcome or target variable is a variable with discrete values. The growth stages of this tree are similar to the ID3 tree, except that it uses the IGR criterion in the development of the tree to classify discrete or continuous features, to prevent overfitting of the tree, and to reduce misclassification error. For different class *K* of feature *X* with an L sample of the dataset and $L_{k}$ samples in *k*th class, the IGR criterion is as follows [27]:

$${\text{IGR}}\left( {L,X} \right) = \frac{{{\text{IG}}\left( {L,X} \right)}}{{ - \mathop \sum \nolimits_{k = 1}^{K} \frac{{\left| {L_{k} } \right|}}{\left| L \right|}log_{2} \left( {\frac{{\left| {L_{k} } \right|}}{\left| L \right|}} \right)}} ,$$

The C5.0 tree is very similar to the C4.5 tree and has fewer disadvantages and is faster and more efficient with memory than C4.5. The C5.0 tree produces smaller decision trees than C4.5. The C5.0 tree considers a lower error rate for new observations, using a smaller set of rules, and automatically drops low-important features. Using the pre-pruning approach, the early growth of the tree is stopped and this strategy causes the training dataset to be classified with high accuracy compared to the C4.5 tree. Thus, the growth stages of the C5.0 tree are such that first, a large tree grows with the training dataset, and then nodes and branches that have little effect on the classification error rate are removed [28].

### Classification and regression tree (CART)

CART abbreviation for Classification and Regression Trees, developed by Breiman et al. CART produces binary trees so that each internal node will have exactly two output edges and the splitting of the internal nodes of the tree is done using entropy or Gini criteria. The generated CART tree can be pruned, and the Cost-Complexity criterion is utilized for the pruning tree, which can consider misclassification costs in tree production, as well as the probability distribution of each node, can be calculated. One of the significant characteristics of CART is its ability to generate regression trees for prediction. In regression trees, the leaves predict a real number and consider divisions where the squared prediction error (squared deviation between predicted and real values) is minimized. Therefore, the prediction on each leaf will be based on the average weight of the node. The entropy measure for a specific feature such as *X* for *C* class, $i = 1, \ldots ,C$, is as follows:

$${\text{Entropy}}\left( X \right) = - \mathop \sum \limits_{i = 1}^{C} \hat{p}_{i} log_{2} \left( {\hat{p}_{i} } \right),$$

where $\hat{p}_{i}$ is the probability of a dataset made up of class i. For different class *C* of feature *X* with an L sample of the dataset, the IG criterion is calculated using entropy as follows [26]:

$${\text{IG}}\left( {L,X} \right) = {\text{Entropy}}\left( L \right) - \mathop \sum \limits_{k = 1}^{K} \frac{{\left| {L_{k} } \right|}}{\left| L \right|}{\text{Entropy}}\left( {L_{k} } \right).$$

Therefore, the Gini index for splitting tree nodes for a subset with N observations will be as follows:

$${\text{Gini}}\left( N \right) = 1 - \mathop \sum \limits_{k = 1}^{K} \hat{p}_{i}^{2} ,$$

If the sample of arbitrary feature *X* wants to be split into two subsets of that node (*X*_1_ and *X*_2_ nodes left and right, respectively), then the Gini index for these two subsets will be as follows:

$${\text{Gini}}_{X} \left( N \right) = \frac{{\left| {N_{1} } \right|}}{\left| N \right|}{\text{Gini}}\left( {X_{1} } \right) + \frac{{\left| {N_{2} } \right|}}{\left| N \right|}{\text{Gini}}\left( {X_{2} } \right),$$

where *N*_1_ and *N*_2_ are the numbers of samples in the left and right nodes and *N* is the total number of samples in the *X* node. Finally, the impurity reduction for feature *X* is shown as:

$$\Delta {\text{Gini}}\left( X \right) = {\text{Gini}}\left( N \right) - {\text{Gini}}_{X} \left( N \right).$$

**Fig. 2:**
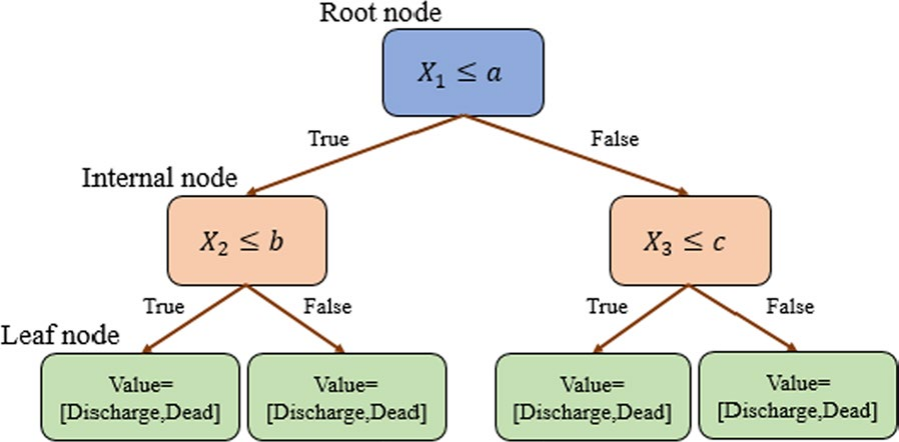
A typical decision tree diagram

The illustration of a typical decision tree diagram with two features *X*_1_, *X*_2_, and *X*_3_ provided in Fig. 2. In this figure, *a*, *b*, and *c* are thresholds for *X*_1_, *X*_2_, and *X*_3_, respectively. These thresholds are obtained by entropy or Gini criteria. For example, if *X*_1_ ≤ *a* is true, then we go to the left side of the tree, otherwise, we go to the right side. This procedure is continued until we reach the leaf nodes. In the leaf nodes, the number of dead and discharged patients are shown given by corresponding branches.

### Performance evaluation metrics

In binary classification, different criteria are introduced to evaluate the performance of decision trees, which show the accuracy of classifying models by comparing true and predicted values. In this study, the confusion matrix is formed of four elements true positive(TP), true negative (TN), false positive (FP), and false negative (FN) in which TP is related to correctly predicted case values, TN is related to correctly predicted no-case values, FP is related to incorrectly predicted case values, and FN is related to incorrectly predicted no-case values. Based on this matrix, Accuracy, Recall, and F1-score indices were calculated and used to evaluate the performance of decision trees. The evaluation criteria introduced are obtained from the following equations that the closer their values are to one, it shows the high accuracy of the models in classification [29]:

$${\text{Accuracy}} = \frac{{{\text{TP}} + {\text{TN}}}}{{{\text{TP}} + {\text{TN}} + {\text{FP}} + {\text{FN}}}} ,$$

$${\text{Precision}} = \frac{{{\text{TP}}}}{{{\text{TP}} + {\text{FP}}}} ,$$

$${\text{Recall}} = {\text{sensitivity}} = \frac{{{\text{TP}}}}{{{\text{TP}} + {\text{FN}}}} ,$$

$${\text{F}}1 - {\text{score}} = \frac{{2 \times {\text{Precision}} \times {\text{Recall}}}}{{{\text{Precision}} + {\text{Recall}}}} .$$

All analysis of this study was done by the scikit-learn module of Python Software version 3.8 and train package of R software version 4.1.2.

## Results

**Fig. 3:**
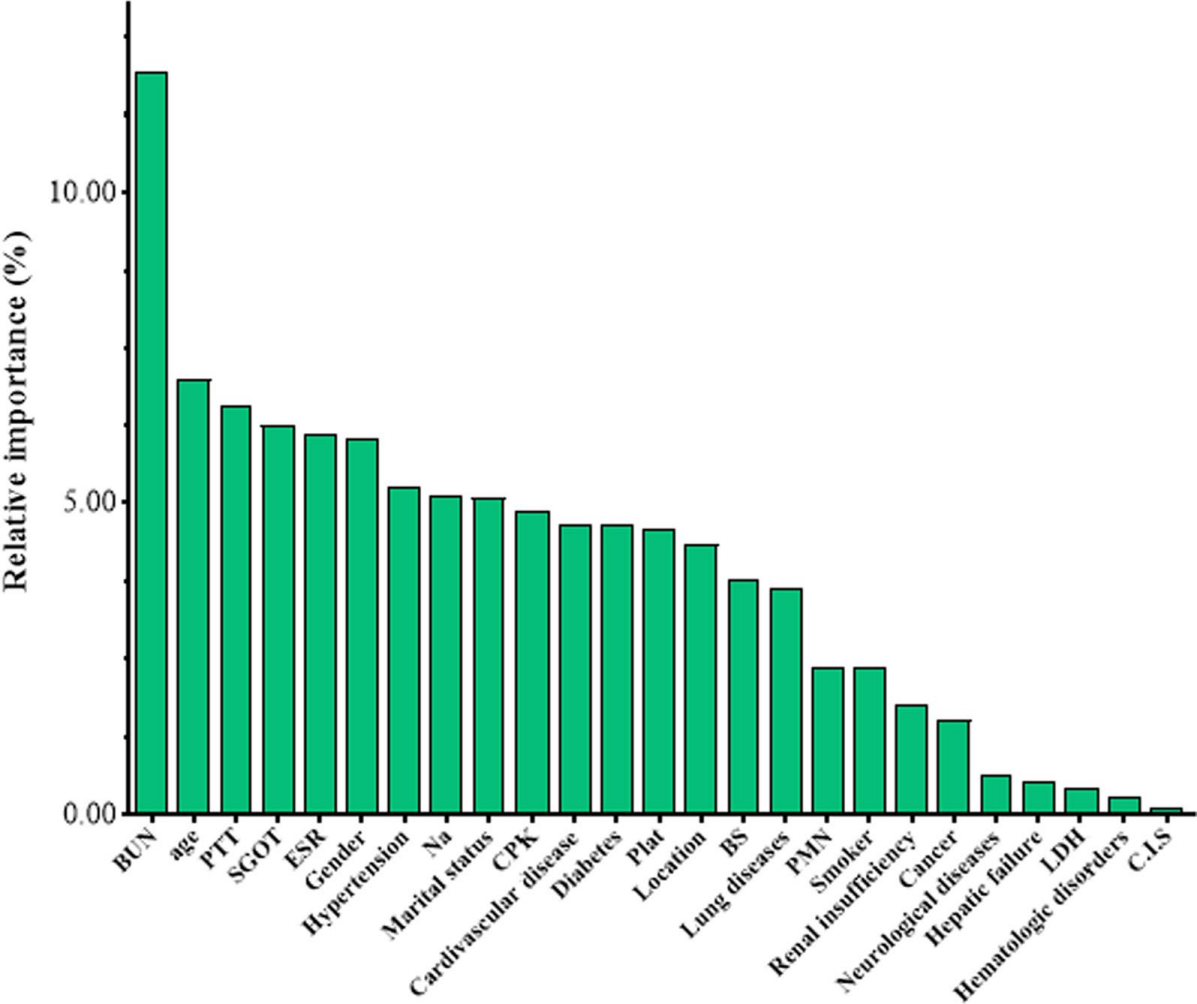
Relative importance features based on RF

In this study, out of 2470 admitted patients, 1853 (75.02%) patients recovered and 617 (24.98%) patients died, of which 111 (18.00%) of deaths were less than 60 years old and 506 (82.00%) were over 60 years old. Also, 347 (56.20%) and 270 (43.80%) of deaths were men and women, respectively. The results of the relative importance of the features for total dataset based on the random forest method in diagnosis the occurrence of death due to COVID-19 are shown in Fig. 3. Also, the descriptive and inference statistics of the studied features with the type of their category and the relative importance values of each feature are reported separately for demographic, clinical, and laboratory findings in Table 1. The results of comparing the demographic, clinical, and laboratory findings features in the two groups of dead and discharged patients showed that the age (*P* < 0.001), hypertension (*P* < 0.001), marital status (*P* < 0.001), cardiovascular disease (*P* < 0.001), diabetes (*P* < 0.001), lung diseases (*P* = 0.004), cancer (*P* < 0.001), Neurological diseases (*P* < 0.001), BUN (*P* < 0.001), PTT (*P* = 0.041), SGOT (*P* < 0.001), Na (*P* < 0.001), CPK (*P* < 0.001), and Plat (*P* < 0.001) features in the two groups were statistically significant and there was a difference between the levels of these features in the two groups of dead and discharged patients.

**Table 1:** Descriptive statistic and importance of each feature in COVID-19 patients

Characteristic	Feature name	Category	Treatment frequency (%)		*P* value*	Relative importance (%)
			Discharge	Dead		
Demographic and clinical	Age	≤ 60	957(51.6)	111(18)	< 0.001	6.99
		> 60	896(48.4)	506(82)		
	Gender	Male	990(53.4)	347(56.2)	0.226	6.05
		Female	863(46.6)	270(43.8)		
	Hypertension	Yes	623(33.6)	283(45.9)	**< 0.001**	5.26
		No	1230(66.4)	334(54.1)		
	Marital status	Married	1552(83.8)	509(82.5)	**< 0.001**	5.07
		Single	109(5.9)	17(2.8)		
		Divorce	15(0.8)	0(0)		
		Other	177(9.6)	91(14.7)		
	Cardiovascular disease	Yes	288(15.5)	171(27.7)	**< 0.001**	4.66
		No	1565(84.5)	446(72.3)		
	Diabetes	Yes	320(17.3)	153(24.8)	**< 0.001**	4.66
		No	1533(82.7)	464(75.2)		
	Location	Urban	1541(83.2)	516(83.6)	0.804	4.34
		Rural	312(16.8)	101(16.4)		
	Lung diseases	Yes	193(10.4)	91(14.7)	**0.004**	3.65
		No	1660(89.6)	526(85.3)		
	Smoker	Yes	140(7.6)	50(8.1)	0.663	2.36
		No	1713(92.4)	567(91.9)		
	Renal insufficiency	Yes	78(4.2)	32(5.2)	0.311	1.77
		No	1775(95.8)	585(94.8)		
	Cancer	Yes	29(1.6)	26(4.2)	**< 0.001**	1.5
		No	1824(98.4)	591(95.8)		
	Neurological diseases	Yes	3(0.2)	11(1.8)	**< 0.001**	0.67
		No	1850(99.8)	606(98.2)		
	Hepatic failure	Yes	16(0.9)	5(0.8)	1	0.57
		No	1837(99.1)	612(99.2)		
	Hematologic disorders	Yes	10(0.5)	6(1)	0.252	0.29
		No	1843(99.5)	611(99)		
	C.I.S	Yes	5(0.3)	1(0.2)	1	0.11
		No	1848(99.7)	616(99.8)		
	Total	–	–	–	–	47.94
Laboratory examination	BUN	≤ 20	1372(74)	202(32.7)	**< 0.001**	11.93
		> 20	481(26)	415(67.3)		
	PTT	30–40	991(53.5)	300(48.6)	**0.041**	6.58
		Other	862(46.5)	317(51.4)		
	SGOT	≤ 45	1378(74.4)	302(48.9)	**< 0.001**	6.25
		> 45	475(25.6)	315(51.1)		
	ESR	≤ 30	698(37.7)	219(35.5)	0.337	6.11
		> 30	1155(62.3)	398(64.5)		
	Na	135–145	1497(80.8)	409(66.3)	**< 0.001**	5.11
		Other	356(19.2)	208(33.7)		
	CPK	25–310	1514(81.7)	413(66.9)	**< 0.001**	4.88
		Other	339(18.3)	204(33.1)		
	Plat	130–400	1578(85.2)	440(71.3)	**< 0.001**	4.59
		Other	275(14.8)	177(28.7)		
	BS	≤ 100	356(19.2)	85(13.8)	**0.002**	3.79
		> 100	1497(80.8)	532(86.2)		
	PMN	< 40	5(0.3)	8(1.3)	0.005	2.36
		40–60	60(3.2)	26(4.2)		
		> 60	1788(96.5)	583(94.5)		
	LDH	100–250	24(1.3)	3(0.5)	0.117	0.46
		Other	1829(98.7)	614(99.5)		
	Total	–	–	–	–	52.06

Significant values are given in bold

*Chi-square test, *C.I.S* compromised immune system, *ESR* erythrocyte sedimentation rate, *BUN* blood urea nitrogen, *BS* blood sugare, *SGOT* serum glutamic-oxaloacetic transaminase, *PTT* partial thromboplastin time, *Plat* platelets, *PMN* polymorphonuclear, *CPK* creatine phosphokinase, *Na* sodium, *LDH* lactate dehydrogenase

According to the results of Table 1, gender and age features of demographic and clinical features and BUN, PTT, SGOT and ESR features of laboratory findings have more than 6% relative importance. The proposed classification models LMT, CART, C5.0, and C4.5 were developed to classify the death event due to COVID-19 for features with a relative importance of more than 6% obtained from the random forest. Recall, Accuracy, and F1-score evaluation criteria for training, test, and total datasets to evaluate the performance of classification models are shown in Table 2.

**Table 2:** Assessing the accuracy of decision tree models in classifying the COVID-19 death

Model	Subset	Confusion matrix				Evaluation metric		
		TP	FP	FN	TN	Recall	F1-score	Accuracy
LMT	Train	1225	299	73	133	0.9437	0.8681	0.7850
	Test	514	130	41	55	0.9261	0.8573	0.7689
	Total	1739	429	113	189	0.9383	0.8650	0.7804
C4.5	Train	1217	284	81	148	0.9376	0.8696	0.7891
	Test	510	120	45	65	0.9189	0.8608	0.7770
	Total	1728	412	125	205	0.9325	0.8655	0.7826
C5.0	Train	1192	276	106	156	0.9183	0.8619	0.7792
	Test	514	130	41	55	0.9261	0.8574	0.7689
	Total	1739	429	114	188	0.9384	0.8649	0.7802
CART	Train	1217	284	81	148	0.9376	0.8696	0.7891
	Test	530	136	25	49	**0.9550**	**0.8681**	**0.7824**
	Total	1739	421	114	196	**0.9385**	**0.8667**	**0.7834**

Significant values are given in bold

**Fig. 4:**
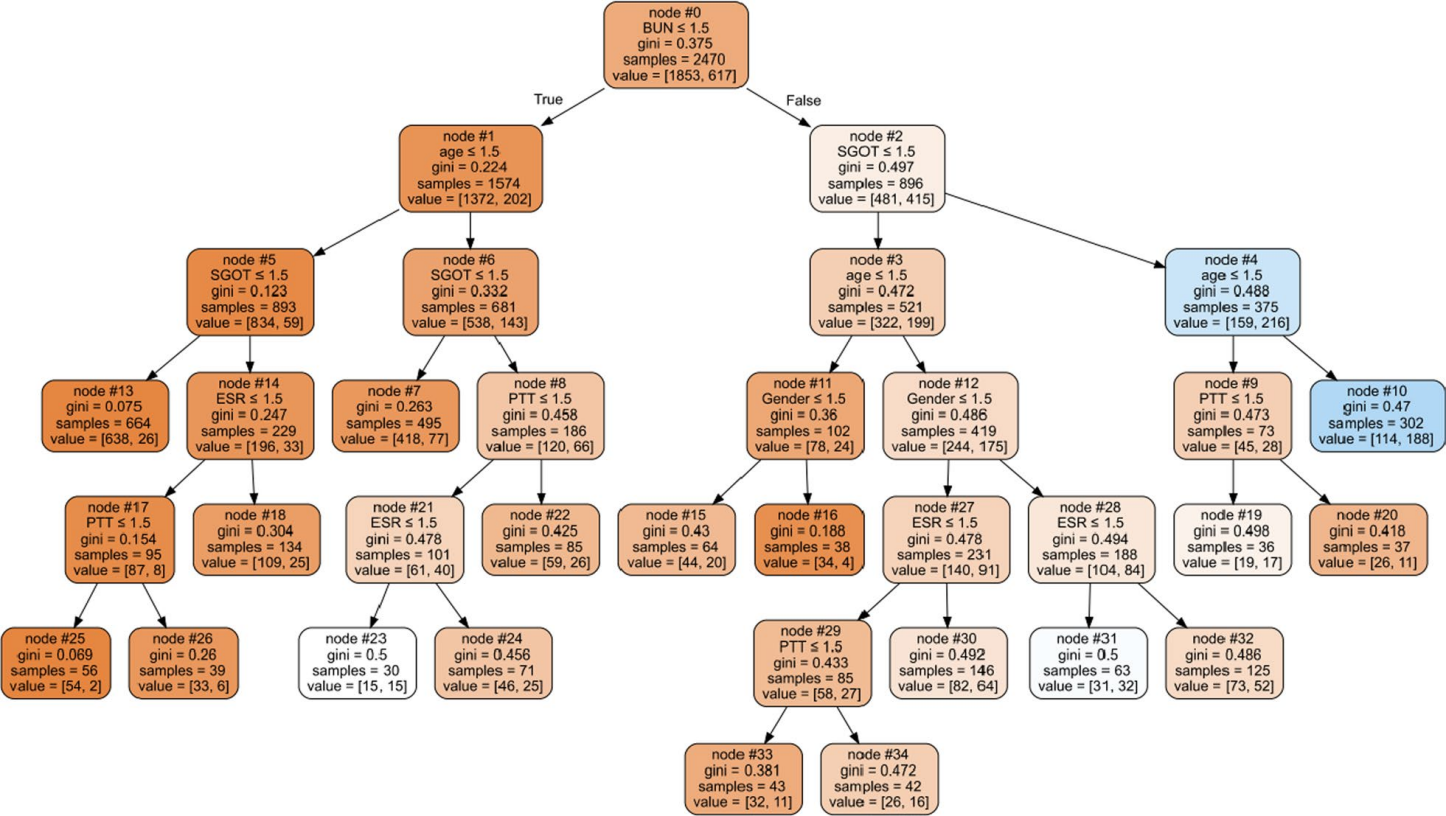
CART flowchart in classifying the COVID-19 death

**Fig. 5:**
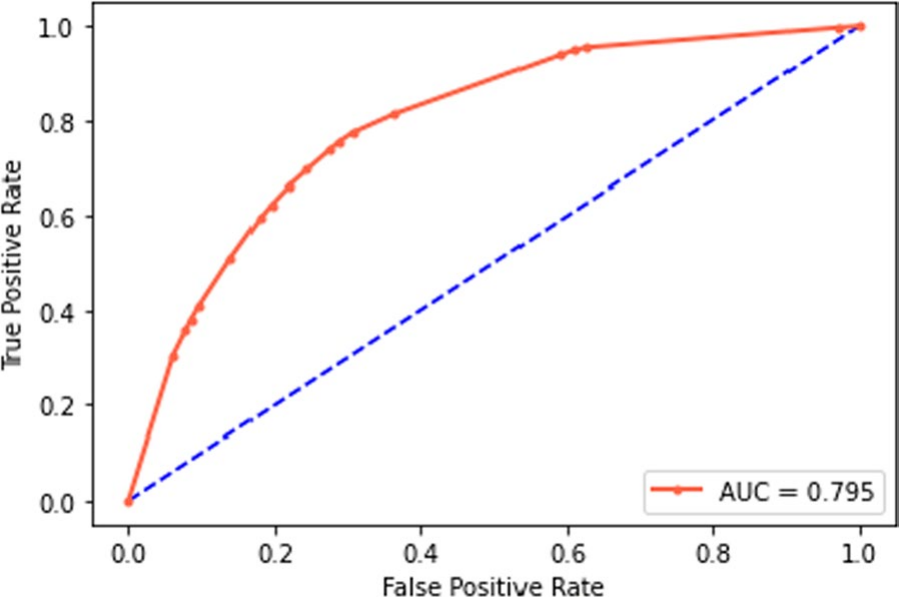
ROC curve for the final CART model

Based on the results of the evaluation criteria, the CART decision tree was selected as the optimal model for classifying the COVID-19 death. The selected CART decision tree flowchart for the gender, age, BUN, PTT, SGOT, and ESR features was drawn based on all the samples of datasets, which are shown in Fig. 4. The ROC curve was plotted to show the performance of the classification accuracy of the final CART model for the total dataset and was shown in Fig. 5.

According to Fig. 4, the interpretability of the tree in diagnosis effective biomarkers based on their importance can be expressed. The developed CART tree consists of 35 nodes, including a root node (zero node), 16 internal nodes, and 18 leaf nodes. For example, nodes zero, 1, 5, and 13 of the first branch of the tree indicate that if an individual had BUN ≤ 1.5 (BUN less than 20), age ≤ 1.5 (age less than 60 years), and SGOT ≤ 1.5 (SGOT Less than 45), the risk of COVID-19 death would be 3.9%. For nodes zero, 2, 4, and 10 of the last branch, the risk of COVID-19 death would be 62.3% for a person with a BUN greater than 20, age above 60 years, and an SGOT greater than 45.

## Discussion

Since the initial diagnosis and identification of some important factors with the more effective on any unknown disease such as COVID-19 is significant for specialists and especially physicians, first, the relative importance of 25 effective features in the probability of COVID-19 death using the random forest method was selected. Then, by selecting important features (above 6%), LMT, C4.5, C5.0, and CART decision tree models were developed based on the training dataset and the performance accuracy of the models in classification was evaluated.

In this study, machine learning methods (decision trees) were used to classify and predict COVID-19 mortality that the most important application of these models is the ability to interpret and predict the future mortality. Therefore, it is principal to use a model that can best classify and predict. The final selected decision tree (CART) can provide the best prediction by optimally classifying the mortality of COVID-19 patients. Deciding to treat such unknown diseases at critical times (golden time) is considered very important for medical professionals. In unknown diseases, such as COVID-19, blood laboratory findings are taken from many patients, which not only increases test time but also test costs or other tests until test results are available. Therefore, the CART decision tree plays an optimal application by prioritizing the most important laboratory findings that are of great importance for optimal decision-making in improving treatment. In most studies with machine learning methods [3, 27], only prediction or classification of some laboratory findings in the occurrence of death of COVID-19 patients has been considered, which has no solution to save treatment time as well as staggering laboratory costs, the most important of which are To be considered, not provided.

In this study, among 25 demographic, clinical, and laboratory findings in the classification of COVID-19 death, demographic and clinical features of age and gender were among the important features with a relative importance of more than 6%. The results of this study have also been shown in studies of Alotaibi et al. and Wang et al. [30, 31]. Also, the BUN, PTT, SGOT, and ESR from laboratory features were among the most important effective features in the COVID-19 death with a relative importance of more than 6% and the results of these findings have also been shown in studies by Cao et al. and Guan et al. [32, 33] which by increasing or decreasing any of these laboratory features from their normal range causes adverse consequences for COVID-19 patients.

Demographic and clinical features of Hypertension, Marital status, Cardiovascular disease, Diabetes, as well as laboratory features of Na, CPK, and Plat are of relative importance above 4.5% in the findings obtained from Table 1, Which can be considered as effective features in the risk of COVID-19 death. In studies by Garg et al. and Nikpouraghdam et al., the features of Hypertension, Cardiovascular disease, and Diabetes, which have been recorded in admitted patients, have been identified as significant features concerning mortality from COVID-19 [34, 35]. In the studies of Peng et al. and Lee et al., the Plat and CPK features were introduced as important features in COVID-19 death [36, 37].

In this study, to show a tree flowchart with easy interpretability for specialists and physicians for prompt treatment decisions, features with more than 6% relative importance were used. For the age, gender, BUN, PTT, SGOT, and ESR features, the LMT, C4.5, C5.0, and CART decision tree models were developed based on the training dataset, which provides accuracy in diagnosing and classifying COVID-19 deaths was 76.89%, 77.7%, 76.89%, and 78.24%, respectively. Also, the F1-score evaluation criteria to show the classification grade were 85.73%, 86.08%, 85.74%, and 86.81%, respectively. Among the developed trees, the CART tree is based on the training dataset with relatively higher accuracy than other trees (Accuracy = 78.24%) and with the classification quality evaluation criterion (F1-score = 86.81%) as the optimal tree in the classification of COVID-19 death was selected. Finally, the decision tree flowchart was drawn by this model to diagnose and identify the patient to decide to provide recovery and provide subsequent treatments.

The risk of COVID-19 death was calculated based on the results flowchart of the final CART tree, and their number of observations was shown in the leaves of the tree in Fig. 2. According to Fig. 2, tree interpretability is helpful for clinicians to prioritize the patient over treatment while making decisions in less time. From the 35 nodes of the tree, which consists of 18 branches from the root node to the leaves, the branch of nodes zero, 1, 5, 14, 17, and 25 indicates the lowest risk of death, namely, if a person is less than 60 years old and BUN ≤ 20 and SGOT ≤ 45 and ESR ≤ 30 and PTT have a range between 30 and 40, then the risk of COVID-19 death is 3.6%. In contrast, the branch of nodes zero, 2, 4, and 10 indicates the highest risk of death, namely, if a person is over 60 years old and has a BUN > 20 and an SGOT > 45, then his risk of COVID-19 death is 62.3%. Also, for women over 60 years old, if BUN > 20, SGOT ≤ 45, and ESR ≤ 30, the risk of COVID-19 death will be 50.8%, as shown in nodes 0, 2, 3, 12, 28, and 31. In the study of Li et al., age and BUN were identified as the most significant influential features in COVID-19 mortality using the Boosting gradient decision tree with accuracy criteria and AUC of 88.9% and 94.1%, respectively [38]. In the study of Bhatia et al., age, BUN, and SGOT features were the most significant features in the severity of COVID-19 mortality, which were obtained by machine learning methods (decision tree) with 79.74% accuracy and 88.3% AUC [38].

Many studies have been performed to classify and predict mortality in COVID-19 patients. In the study by Yadaw et al. and de Moraes et al., they examined the effects of laboratory findings on the prediction of COVID-19 mortality using machine learning methods, in which the performance of predicting mortality in their study based on the AUC index was 91% and 85%, respectively [14, 15]. It should be noted that despite the high value of these indices, there is no interpretation of the effect of features on the outcome.

One of the strengths of this study is using a tree-based models that transforms the data into a tree representation and could be easily interpreted and understood. These non-parametric models can efficiently deal with large and complicated datasets without distributional assumptions. In fact, when the dataset was small, these models suffer from overfitting or underfitting. In this study we overcome this issue by using the quite large dataset. As in other studies, the researchers of this study faced with some limitations. In addition to the features used in this study, there are some features such as ALP and ALT (or SGPT) due to the lack of information in the patient file or the presence of a lot of missing in these features do not consider in the analysis process. Also, in this study, researchers had access to only two centers in Hamadan, Iran, which could have been generalized with more power and confidence if all cities in the province were considered. Since many feature selection methods have been introduced, applying different feature selection methods and comparing their results with each other from the point of view of selected features, and examining the performance of these methods in subsequent analyzes such as classification can provide more detailed information for researchers.

## Conclusion

Finding a highly accurate and qualified model for interpreting the classification of an outcome that is considered clinically very important, at all stages, including treatment and immediate decision-making, is crucial. Therefore, the use of high-accuracy machine learning methods with simple interpretability, such as the CART decision tree, can help in diagnosing, classifying, and prioritizing the factors influencing in death of COVID-19 patients, as well as other diseases.

## Data Availability

The dataset used for analysis during the current study are not publicly available due to restrictions related to our internal review board policy. However, the dataset is available from the corresponding author (Ali Reza Soltanian) on reasonable request.

## Abbreviations

LMT: Logistic model tree
CART: Classification and regression tree
ROC: Receiver operating characteristic
AUC: Area under the curve
BUN: Blood urea nitrogen
PTT: Partial thromboplastin time
SGOT: Serum glutamic-oxaloacetic transaminase
ESR: Erythrocyte sedimentation rate
IGR: Information gain ratio
ESR: Erythrocyte sedimentation rate
BUN: Blood urea nitrogen
BS: Blood sugar
CPK: Creatine kinase
PTT: Thromboplastin or partial thromboplastin time
Plat: Platelets
Na: Sodium
LDH: Lactate dehydrogenase
PMN: Polymorphonuclear
ID3: Iterative Dichotomiser 3
TP: True positive
TN: True negative
FP: False positive
FN: False negative
C.I.S: Compromised immune system
ALP: Alkaline phosphatase
ALT: Alanine transaminase
